# iArm: Design an Educational Robotic Arm Kit for Inspiring Students’ Computational Thinking

**DOI:** 10.3390/s22082957

**Published:** 2022-04-12

**Authors:** Chengze Zeng, Hong Zhou, Weiwei Ye, Xiaoqing Gu

**Affiliations:** 1 Department of Education Information Technology, East China Normal University, Shanghai 200062, China; cz.zeng@outlook.com (C.Z.); 51204108022@stu.ecnu.edu.cn (W.Y.); xqgu@ses.ecnu.edu.cn (X.G.); 2 Shanghai Engineering Research Center of Digital Education Equipment, East China Normal University, Shanghai 200062, China

**Keywords:** educational robotics, robotic arm kit, curriculum design, computational thinking

## Abstract

Educational robotics is an effective carrier of information technology education, making its way into classrooms. However, the design of the educational robotic arm kit and the study on the effect of robotic arms on students’ thinking literacy remain to be completed. In this paper, iArm, a 6-DOF robotic arm consisting of a drive chassis, an arm body, and end tools, is presented. Its auxiliary modules, including the vision module and conveyor belt, and the curriculum targeting students’ computational thinking are also developed to refine the current educational robotic arm kit. Furthermore, to explore the effectiveness of the iArm kit, thirteen high school students participated in the semester-long curriculum, completed assigned projects, and filled out the pre-test and post-test scales. By formative and summative evaluation, the result shows that the iArm kit effectively enhanced students’ computational thinking.

## 1. Introduction

### 1.1. Background

In recent years, the tide of artificial intelligence has gradually brought robots into the public and the development and application of educational robotics have been increasingly common. Educational Robotics (ER) refers to the technology of constructing and programming a robot as an educational tool [1]. It integrates theories and technologies not limited to engineering, electrical engineering, computer science, and pedagogy.

Along with the popularization of ER, an increasing number of teams pay heed to the construction and applications of educational robots. The robots in the educational market mainly focus on mobile robots such as Thymio II [2] and Turtlebot 3 [3]. But articulated robots and humanoid robots also have a place. Educational robots cover a wide range of education levels from K-12 to higher education, and some robots share certain characteristics including the focus on assistive functions like buttons, grayscale sensors, and cameras. Some robots are equipped with client software, and some are capable of Python and ROS programming [4].

In addition to constructing educational robots, the application effect deserves to be explored further. With the advent of LEGO Mindstorms products in classrooms, students combined engineering concepts with practice and explored notions of robot design [5]. In line with the concept of constructionism that being involved in the creation of concrete artefacts can result in a more efficacious learning process as proposed by Papert [6], learners are stimulated to be more technologically literate through the construction and interaction with robots. They apply what they have learned to manipulate concrete objects and solve concrete problems and, as a result, gain a deeper understanding of the construction logic and the function of different parts of robots. 

In addition to the assembly and operation of hardware and device, ER has shown its potential in subjects beyond robotics [7]. Educational robotics plays a key role in the education of science, technology, engineering, and mathematics (STEM) [8]. Through robotics, learners are provided with the opportunity to hone their programming language and dabble in kinematics, computer vision, and more. Long-term intervention can lead to a significant effect in STEM learning and short-term intervention, and though without the learning advantages gained over the long term, has the potential to improve their motivation and attitude towards STEM-related subjects [9]. Also, ER can further foster the enhancement of abilities that are conducive to learning performance such as computational thinking [10], problem-solving skills, and creative thinking [11].

In response to the needs of the technological society and recognition of the advantages of ER, schools are already offering robotics-related courses to improve students’ STEM literacy and establish robotics departments. Top American universities in technology like Carnegie Mellon University and Stanford, have many courses and programs in robotics to cultivate STEM graduates [12]. In Argentina, the University of Buenos Aires develops robotics-related courses, talks, and exhibitions [13]. And in Japan, Ritsumeikan University established the first Department of Robotics in 1996 [14]. Other than robotics courses for college students, courses for high school students have been developed to provide access to robotics hardware and robot control [15]. 

The constant attention comes not just from schools, but also from international institutions. To maximize the advantages of ER applications, numerous robotics competitions targeting different educational levels are hosted. VEX Robotics Competition and some other international competitions are offered by The Robotics Education and Competition Foundation. The Asia-Pacific Broadcast Union Robot Contest, Boosting Engineering, Science, and Technology, and RoboParty are respectively held in Asia, the USA, and Europe [16]. In South Africa, the World Robotics Olympiad was hosted, and it encouraged autonomy and collaboration among the participants [2].

### 1.2. Purpose and Target of This Study

Currently, robotic arms in the field of education are mainly commercial ones. They are not suitable for wide application in education due to their high price and closed source, etc. Therefore, the purpose of the study is to develop a low-cost, open-source robotic arm kit for students to use. We aim to educate students on both robotic arms and programming skills to cultivate students’ computational thinking. They can learn a series of skills, such as algorithm design and programming skills, and develop their thinking literacy, including creative thinking, critical thinking, etc., by completing the projects of our curriculum. 

The educational robotic arm kit comprises iArm, which is a 6-degree-of-freedom (DOF) robotic arm, and its ancillary tools include a gripper, a vacuum pump and a conveyor belt, and an arm-based curriculum. The curriculum details how to complete a series of projects using the robotic arm and its tools.

The main target users for the robotic arm kit are high school students with certain mathematical foundations and logical reasoning abilities. The kit can be applied in high school information technology courses or extension courses for students interested in learning about robotics or programming.

### 1.3. Contribution

The study aims to provide students with tools that allow them to learn robotics and programming skills and to inspire their computational thinking. The contributions of the paper are as follows:

1. We designed a low-cost, open-source robotic arm and ancillary tools including a vacuum pump and conveyor belt for inspiring students’ computational thinking.
2. We developed a three-stage iArm-based curriculum for students.
3. We detailed three experiments in the curriculum to show how to cultivate students’ computational thinking by using this kit.
4. We verified that the students computational thinking was improved during the curriculum in five dimensions including creative thinking, critical thinking, algorithmic thinking, problem-solving thinking, and cooperative thinking.

Current educational robotic arm kits on the market are too expensive to be widely used in education. They are also “closed source”, which is not conducive for students to further develop the arm by learning the internal code. However, we not only refine the hardware devices but also provide courses to facilitate teaching. Moreover, iArm is low-cost and open-source, so students can learn the internal code and even develop additional functions. Furthermore, few studies have explored the effect of robotic arm courses on the cultivation of computational thinking, so our study can provide a reference for other studies.

### 1.4. Paper Organization

The remaining sections of the paper are structured as follows. In Section 2 we review the current research relevant to educational robotics and computational thinking. In Section 3 we introduce the hardware and software part of iArm. Section 4 presents the curriculum design and three detailed experiments for improving computational thinking. It also assesses the effect of the curriculum on students’ computational thinking from formative and summative evaluation and, finally, Section 5 concludes the article.

## 2. Related Work

Many issues concerning educational robotics and computational thinking have been addressed. The related works are described below and cover the aspects of educational robotic arms, the educational applications of arms, and computational thinking.

### 2.1. Educational Robotic Arm

Educational robots are mainly mobile robots, while articulated robots have also been developed for education. Common robotic arms are multi-DOF arms fitted with an arm gripper. The cost of robots is a consideration for many researchers. Cocota et al. [17] developed a 4 + 1 DOF serial manipulator with a two-finger gripper made with acrylic. With the low cost of 150 dollars, it served as a challenge to motivate students’ ability to design and construct the manipulator. Also, it can be used by students to solve forward and inverse kinematic problems. Components of WayBotDu developed by Adinandra et al. [18] were all available in local shops or accessible online.

Researchers have gradually adjusted the structure of the arm over time. Krasňanský et al. [19] designed a robotic arm with 6 + 1 DOF and developed convenient electronic modules including a Hitec joint module, a Tonegava joint module, and a gripper module. Cocota et al. [20] improved the sampling of the average angular velocity of the joints and the kinematic control of the trajectory position of the previous robot. The manipulator robot of the SCARA type [21] that was invented by Neto et al. modified the traditional fixed-based structure to develop horizontal linear motion. The current-efficient power supply of the 5 DOF manipulator made by Lobur et al. [22] requires no fans for cooling. The basic design of robotic arms is being gradually refined, with numerous studies underway.

Furthermore, auxiliary functions were attached to robots. The articulated robot developed by Manzoor et al. [23] was equipped with a camera to identify the properties of the objects in the workspace and a force sensor fixed at the end-effector to distinguish objects with different stiffness. The wireless function was added through a microcontroller using Arduino Yun, and the segments of the arm were printed on a 3D printer [24]. Then Hudy et al. [22] developed the capacity of executing sequences in a loop in embedded system applications and Trehan et al. [25] developed the joint control function in ROS for KOBOKER. Though current articulated robots are well-designed, more refinements remain to be made.

Among commercial robotic arms, Magician Lite, a 4 DOF robotic arm, is produced by Dobot. Its end tools include a pen holder, a suction cup, and a soft gripper [26]. Although the extensibility of robotic arms is considered as much as possible, due to 4 DOF and structural design, Magician Lite can only work on the base level. Also, the commercial robotic arm is too expensive for general application and the platform is not open source for purchasers. Thus, the team developed a 6 DOF robotic arm that is able to work on curved surfaces with about a quarter of the development cost of Dobot. And the internal program is open source to students for secondary development.

### 2.2. Educational Applications of Robotic Arm

Applications of robotic arms in education are mainly concentrated in higher education. Robotics involves many technical subjects and can be widely used. Therefore, robot instruction at this stage is characterized by a combination of education and innovation [27]. The Department of Robotics established in 1996 at Ritsumeikan University integrated robotic arms into practical robotics education. The course included robot experiments using 3-DOF and 6-DOF manipulators and robot construction [14]. Subsequently, in the course “Introduction to Robotics” offered by Harvard University, open-architecture industrial arms were introduced so that students can learn forward and inverse kinematics, velocity kinematics, path planning, and computer vision in turn [28]. Then many researchers have made changes to the robotics curriculum. Project-based learning methodology [29], challenge-based learning methodology [30], and the interactive learning environment [31] were introduced into courses. Also, robotic arms were used in other courses like hardware description language teaching [32]. Applications of educational robotic arms positively resulted in higher motivation, better communication skills [30], and the development of transversal skills [20]. 

In addition to higher education, robotic arms were also applied in other education stages. Educational robotics is commonly associated with STEM education [33]. Chu et al. [34] investigated a high school robotic arm educational competition and found that students performed integrated STEM capability and unique creativity. Nevertheless, students’ attitudes towards STEM showed no significant difference for uncertain factors. Verner et al. [35] assessed students’ learning effects after several weeks of courses and found that students acquired an initial understanding of concepts and showed higher interest in intelligent robotics. Also, attitudes towards STEM are influenced by the socioeconomic status of the students [36]. Other than secondary education, in special education, robotic arms can facilitate classroom participation, expressive language, and interest in robot tasks for children with disabilities [37]. Educational robotic arms have been developed to a certain degree, while their educational kit design is still a work in progress. 

### 2.3. Computational Thinking

Computational thinking, defined by Wing, involves solving problems, designing systems, and understanding human behavior by drawing fundamental concepts of computer science [38]. The International Society for Technology in Education (ISTE) believes that all students should graduate from high school with computational thinking, and that it should be integrated into formal education [39]. ISTE categorizes computational thinking as creative thinking, critical thinking, algorithmic thinking, problem-solving thinking, and cooperative thinking. Creative thinking refers to the creation of unusual ideas, critical thinking helps students choose the optimal solution, and students are required to summarize the parts of the problem, thus making problem-solving thinking important. Algorithmic thinking presents students’ logic to solve problems. And when students are completing difficult projects, they are required to cooperate and communicate well. The interaction of the five thinking skills leads to computational thinking. China regards computational thinking as one of the core qualities of information technology in senior high schools [40]. High school students should learn how to exert computational thinking on problem analysis, abstraction, modeling, and designing systematic solutions.

The cultivation of computational thinking is mainly through computer programming including traditional programming languages like Java and visual programming platforms like Scratch [41]. Scratch is believed to have the potential to teach computational thinking skills and facilitate creativity and problem-solving thinking [42]. Also, Computer Science Unplugged makes computer science accessible to K-12 students to cultivate computational thinking [43]. Educational robotics have also been applied in education settings to foster computational thinking [44]. Various tools have been studied for their potential to cultivate computational thinking. 

However, few studies have focused on the educational effects on computational thinking of robotic arms, so this study explores the educational significance of iArm on computational thinking from the perspectives of creative thinking, critical thinking, algorithmic thinking, problem-solving thinking, and cooperative thinking to fill in the gaps in the field.

## 3. iArm Design

### 3.1. Overview of iArm

**Figure 1:**
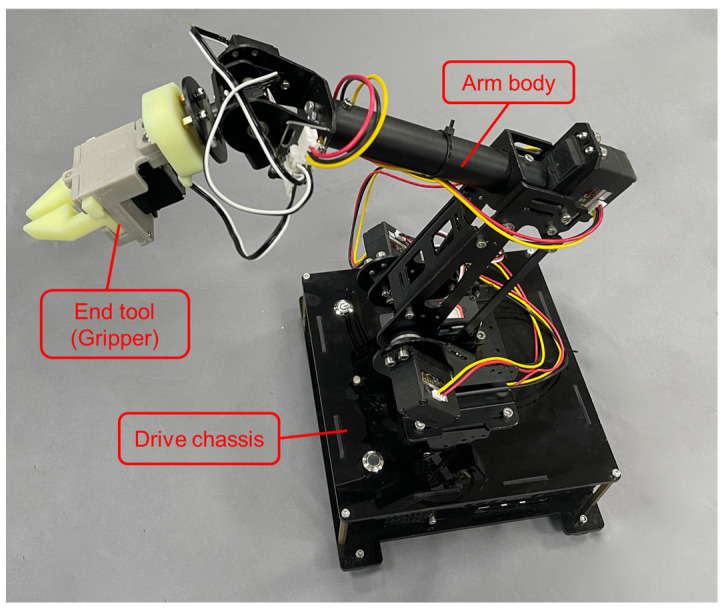
iArm and its components.

iArm is a 6-DOF robotic arm with three main components, namely a drive chassis, an arm body, and end tools, as shown in Figure 1. It features a high degree of bionics, a well-considered drive design, and a variety of tools including grippers, vacuum pumps, conveyor belts, etc. It can be programmed to clip and carry small objects based on kinematics or applied to a machine learning-based vision pick. The purpose of iArm is to teach students artificial intelligence technology with specific cases, helping them deeply understand the process of perception, operation, storage, and expression in machines to improve students’ computational thinking and cultivate their innovative spirit and problem-solving ability.

The iArm is based on the Robot Operating System (ROS), which is not just an operating system but a software framework providing typical robot activity modules such as object recognition and motion planning [45]. Due to the high extensibility of ROS, users can further extend the functionality of iArm based on their needs, such as the use of sensors and conveyor belts and multi-arm controls. 

**Figure 2:**
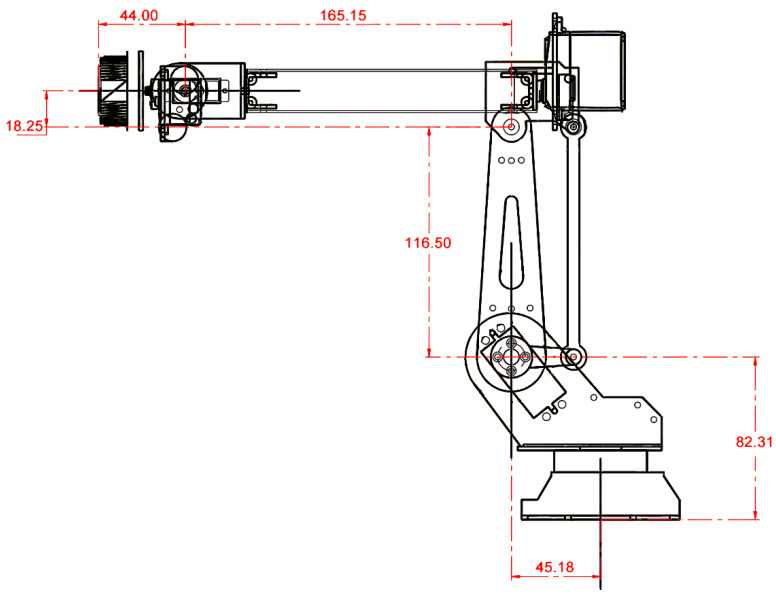
URDF model of iArm.

Also, the team designed, simulated, and adjusted the precision of the robotic arm through the Unified Robot Description Format (URDF) parameter, so that the precision of iArm was gradually improved. URDF is an XML format used to represent the structure of the robot and its potential action in commercial simulators [46]. Table 1 is the standard D-H parameter table and Figure 2 is the URDF model of iArm. Users are allowed to perform simulations in Gazebo and take a preliminary look at iArm’s movements in rviz, a 3D visualizer for visualizing robots, workspace, and sensor data in ROS.

### 3.2. iArm Hardware

The iArm consists of three main components, namely a drive chassis, an arm body, and end tools. In addition, conveyor belts and visual modules including the camera, workspace panel, and camera stand are equipped for users who can tailor the use of iArm to their needs.

#### 3.2.1. Drive Chassis

**Figure 3:**
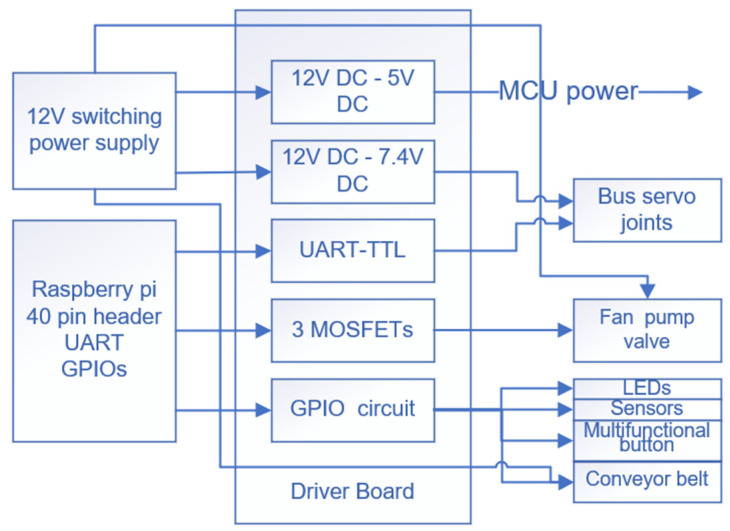
Schematic diagram of the driver board.

The drive chassis is the basis of the iArm control, which contains the main controller of iArm, namely a Raspberry Pi 4 Model B. And it is connected to the drive board via a Raspberry Pi pin header. As shown in Figure 3, the driver board includes 12 V to 7.4 V and 12 V to 5 V two channels of the DC-DC conversion part, three channels of the MOSFET switch, a fan control, a UART to TTL part, and a multi-functional button control part. Two 4010 12 V fans, a 12 V 75 W switching power supply, an I/O panel, and a power switch are linked to the driver board. Among them, two fans are divided into two modes of continuous rotation and conditional rotation, respectively, to fully consider the heat dissipation of the drive chassis.

To enrich the auxiliary functions of iArm, a multi-functional button and a three-color LED are designed on the upper plate of the chassis, which are respectively used to convert the arm into different operation modes and mark the status of iArm. An I/O panel is installed on the rear plate for connecting other tools, including servos, sensors, conveyor belts, and vacuum pumps.

#### 3.2.2. How iArm Works

When using iArm, the user applies 220 V AC power to the AC socket, which is converted to 12 V DC power through the switching power supply. Then 12 V DC is converted to 7.4 V and 5 V, respectively, through the power module of the driver board to supply power to the servos and the main control, each of which can output 3A peak current. Three channels of the MOSFET switch control the external devices, one channel of UART to TTL realizes the control of the serial interface to TTL asynchronous half-duplex bus servo, and the interface at the I/O panel is directly connected to the GPIO port of the main control, which is used to control the LED and the stepper motor driver.

After the power supply, the first to the sixth servo in series respectively drives each joint of the arm body to rotate and the seventh servo drives the end tool.

#### 3.2.3. Vacuum Pump

**Figure 4:**
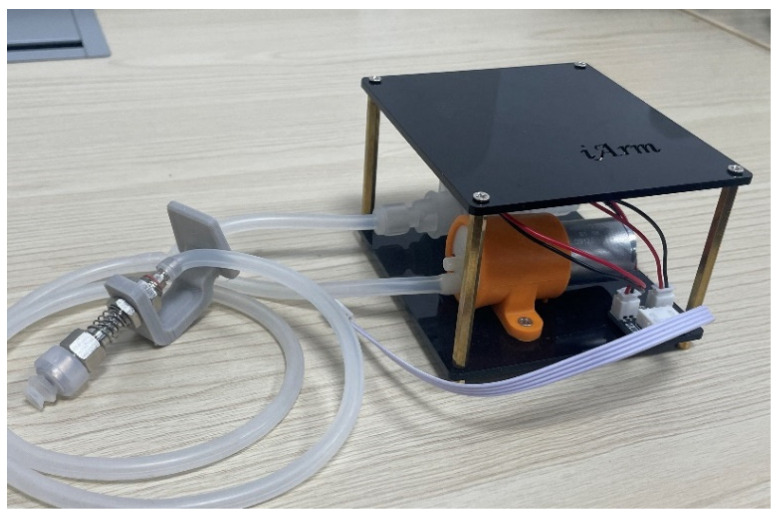
Vacuum pump.

As shown in Figure 4, the vacuum pump is one of the end tools for iArm, which can be installed into the hand of the arm body. To assemble the device, it is connected to the solenoid valve through a suction cup. Then the suction cup is plugged into the hose. While in use, the vacuum pump is linked to the I/O panel on the rear panel of the iArm drive chassis through a 4-pin Dupont line.

#### 3.2.4. Conveyor Belt

**Figure 5:**
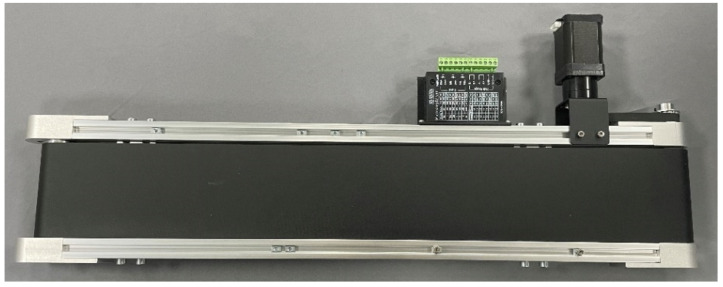
Conveyor belt.

A conveyor belt is shown in Figure 5. The core device of the conveyor belt is a TB6600 stepper motor driver for controlling the two-phase stepping motor, which allows users to set its micro step and output current [47]. Due to the motor driver, users can adjust the rotation direction and speed of the stepper motor. After assembling the belt, we connected the stepper motor driver to the stepper motor and linked it to the I/O panel through a 4-pin Dupont line to enable the user to control the belt by calling the API or through the client.

### 3.3. iArm Software

The software of iArm is partially modified based on the Niryo One, whose stack package is open source to the public. Packages of the Niryo One ROS stack [48] help developers gradually improve the functions of the robot from the bottom of the hardware layer to the control layer, motion planning layer, command & user interface layer, and finally to the external communication layer. Among them, some of the modifications we made were as follows.

**Figure 6:**
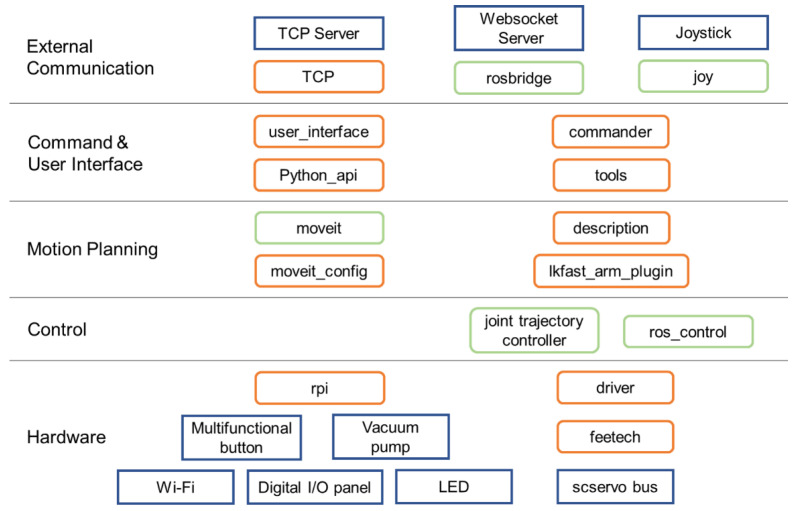
ROS stack.

First, Niryo One’s joints use four stepper motors and three servos, while iArm uses seven serial servos. Therefore, we modified the driver package to support our servos. In addition, according to the driver board designed by the team, we modified the rpi package to support our peripherals and hardware, such as sensors, vacuum pump, LED control, etc. Our ROS stack is shown in Figure 6.

## 4. Results and Discussion: iArm in Education

### 4.1. Curriculum Schedule

The iArm-based curriculum is designed for high school students. It requires students to have a certain mathematical foundation, logical reasoning ability, and an interest in learning robotics and programming. Also, it does not require students to have programming ability in advance. The curriculum includes 16 classes and 10 projects and lasts for one semester. The detailed curriculum schedule is distributed into two parts, namely curriculum objectives and curriculum content. The course content includes course activities and corresponding computational thinking ability.

#### 4.1.1. Curriculum Objectives

Computational thinking development objectives are divided into five dimensions: problem abstraction, algorithm design, iteration optimization, test and correction, and generalization and application. The specific objectives are shown in Table 2.

#### 4.1.2. Curriculum Content

Stage: Preliminary stage

Activity

**Project 1**: “Drive” iArm: Move the Robotic Arm with the Controller

Content:

(a) The development and application of robotic arms
(b) The concept of robotic arm degrees of freedom.
(c) The hardware and client of iArm.

Problem Setting: 

How do the joints of iArm rotate and interact with each other?

Experiment: Move iArm with a controller.

**Project 2**: Basic Movement of iArm: Graphical Programming

Content:

(a) The status information of iArm
(b) Coordinates of joints and end tools.
(c) Learning mode and graphical programming

Problem Setting: 

How to make iArm move on a horizontal plane?

Experiment:

(a) Perform linear motion through the endpoint system.
(b) Perform oblique motion, making iArm write “7” or “Z” on the desktop.

Dimensions of Computational Thinking:

Students think of the way to make iArm move horizontally. The project focuses on training algorithmic thinking and problem-solving thinking.

**Project 3**: Basic Movement of iArm: Linux Control

Content:

(a) Python programming
(b) iArm Control in Linux

Problem Setting: 

How to make iArm movement as smooth as possible?

Experiment: Control iArm to move along x, y, and z axes.

Dimensions of Computational Thinking:

Students think about how to make iArm move smoothly and optimize their algorithms, such as by using interpolation, thus improving their creative thinking, critical thinking, algorithmic thinking, and problem-solving thinking.

Computational thinking skills

Abstraction

Task: Perform linear and oblique motion.

Problem decomposition: 

The way to move in a straight line along the x, y, and z axes is to keep the coordinates on both axes constant and change the coordinates on one axis.

Algorithm

Data analysis: 

Measure the motion range of x, y, and z coordinate axes of iArm, and perform the linear or oblique motion of iArm within the range.

Function call:

(a) move_pose (x, y, z, roll, pitch, yaw)
(b) shift_pose (axis, values)

Optimization

Program structure:

(a) Sequential structure
(b) Loop structure

Test

Run the program/ use the controller, observe the movement of iArm, and modify the code according to the actual error.

Generalization

Analyze the pros and cons of the two programming methods for better performance in subsequent experiments.

Stage: Intermediate stage

Activity

**Project 4**: Robotic Arm Palletizing

Content:

(a) Industrial applications of palletizing robots
(b) Analysis of iArm palletizing process
(c) Function call: pick/place from the current position

Problem Setting: 

What logical structure and clamping order is the most efficient?

Experiment: Robotic Arm Palletizing

Dimensions of Computational Thinking:

Students collaborate to design the optimal algorithm for clip placement to train all five dimensions of computational thinking.

Computational thinking skills

Abstraction

Task: Robotic arm palletizing

Keep the gripper vertically down and stack the blocks at places 1, 2, and 3 to place 4 in turn.

Problem decomposition:

(a) How to move the block from place 1 to place 4?
(b) How to move the blocks at places 1, 2, and 3 to place 4?
(c) How to derive the coordinates at places 2, 3, and 4 from the coordinate at place 1?
(d) Analyze the process of palletizing (pick and place).

Algorithm

Data analysis: 

Analyze the process of palletizing and measure the distance between each area.

Function call:

(a) pick_from_pose (x, y, z, roll, pitch, yaw)
(b) place_from_pose (x, y, z, roll, pitch, yaw)

Optimization

Program structure:

(a) Sequential structure
(b) Loop structure

Test

Run the program, observe the palletizing, and modify the code according to the actual error.

Generalization

Analyze the effect of palletizing and summarize the difficulties and solutions during the experiment.

**Project 5**: Robotic Arm Painting and Writing

Content:

(a) Applications of writing robots
(b) Analysis of iArm writing
(c) Function call: pose/axis move

Problem Setting: 

What logical structure and movement order is optimal?

Experiment: Robotic arm painting and writing

Dimensions of Computational Thinking:

Students collaborate to design the optimal algorithm to write the character. They apply their logical foundation to the designing and optimization of the algorithm. The experiment cultivates students’ computational thinking in all five thinking dimensions.

Computational Thinking Skills

Abstraction

Task: Robotic arm painting and writing

Problem decomposition:

Start and stop the strokes between different strokes.

Start the stroke: Raise the nib away from the paper, that is, the z coordinate value increases.

Drop the stroke: Drop the nib down to the paper, that is, the z coordinate value decreases.

(a) How to draw on the XY plane?
(b) How to draw a line?
(c) How to increase the “start” and “drop” actions?
(d) How to draw two lines?
(e) Thinking: How to make iArm dip ink automatically?

Algorithm

Data analysis: 

Measure z-coordinate values at the start and end of the stroke.

Function call:

(a) move_pose (x, y, z, roll, pitch, yaw)
(b) shift_pose (axis, values)

Optimization

Program structure:

(a) Sequential structure
(b) Loop structure

Test

Run the program, observe the painting, and modify the code according to the actual error.

Generalization

Analyze the effect of writing and painting and summarize the difficulties and solutions during the experiment.

Stage: Advanced stage

Activity

**Project 6**: Computer Vision: Color-based Pick

Content:

(a) Computer color system
(b) iArm vision pick

Problem Setting: 

How to call functions to distinguish colors and clip blocks through iArm?

Experiment: 

Clip blocks with different colors in the workspace to the corresponding color area.

Dimensions of Computational Thinking:

Students design algorithms and call functions to distinguish colors. Their algorithmic thinking and problem-solving thinking are greatly improved.

**Project 7**: Machine Learning: Candy Pick

Content:

(a) Machine learning
(b) iArm candy pick principle

Problem Setting: 

How can machine learning be used to identify candies?

Experiment: 

Clip different candies in the workspace to the corresponding area.

Dimensions of Computational Thinking:

This project needs students to discuss fully how to design the program, and gradually realize the function. This project mainly requires students’ problem-solving and algorithmic thinking, and it also needs the assistance of the other three kinds of thinking to complete the task.

**Project 8**: Multi-arm Collaborative Synthesis Experiment

Content:

(a) Robot Operating System (ROS)
(b) Application mode of Industry 4.0
(c) Sensor-related knowledge

Problem Setting: 

How to control two iArm meanwhile and return sensors’ results to the iArm?

Experiment: Multi-arm collaborative synthesis experiment

Dimensions of Computational Thinking:

Students learn to summarize the needs and realize the goals step by step, including control, recognition, value transmission, etc., which is a challenge to students’ algorithmic thinking. Students’ computational thinking will be improved in this project.

**Project 9**: Matlab Simulation

Content:

(a) Matlab modeling and simulation
(b) D-H parameter of a 6-DOF robotic arm

Problem Setting: 

How to establish a 6-DOF robotic arm model according to the D-H parameter table and related functions?

Experiment: Establish a 6-DOF robotic arm model in Matlab.

Dimensions of Computational Thinking:

Students should understand the relationship between the D-H parameter table and the structure of the robotic arm and create each joint gradually. During the project, their creative thinking, critical thinking, algorithmic thinking, and problem-solving thinking will be improved.

**Project 10**: Chess-playing Robot

Content:

(a) Historical development of man-machine chess
(b) iArm chess principle

Problem Setting: 

How does iArm choose the best step based on the situation?

Experiment: Play chess with iArm.

Dimensions of Computational Thinking:

Students connect iArm with the vision module and design chess algorithms, which they can refer to current algorithms online and optimize them. During the task, students cultivate their computational thinking skills.

Computational thinking skills

Abstraction

Task: iArm vision module

Realize artificial intelligence robotic arm through camera and sensors.

Problem decomposition:

(a) How do robotic arms distinguish wood blocks with different colors?
(b) How do robotic arms distinguish different types of candy?
(c) How do robotic arms cooperate with intelligent cars?
(d) How do robotic arms play chess with humans?

Algorithm

Data analysis: 

Measure the coordinates of the corresponding area.

Function call: 

Vision_pick (workspace, height_offset, shape, color)

Optimization

Program structure:

(a) Sequential structure
(b) Loop structure
(c) Selection structure

Test

Run the program, observe the effects of vision pick, candy pick, chess, arm-vehicle coordination, modeling and simulation, and modify the code according to the actual error.

Generalization

Analyze the effects of vision pick, candy pick, chess, arm-vehicle coordination, modeling and simulation, and summarize the difficulties and solutions during the experiment.

### 4.2. Experiments of iArm

The three experiments in this course are as follows, including details of the experiments and how students can improve their computational thinking through the three experiments.

#### 4.2.1. Experiment I: Vision Pick

Experiment Content

**Figure 7:**
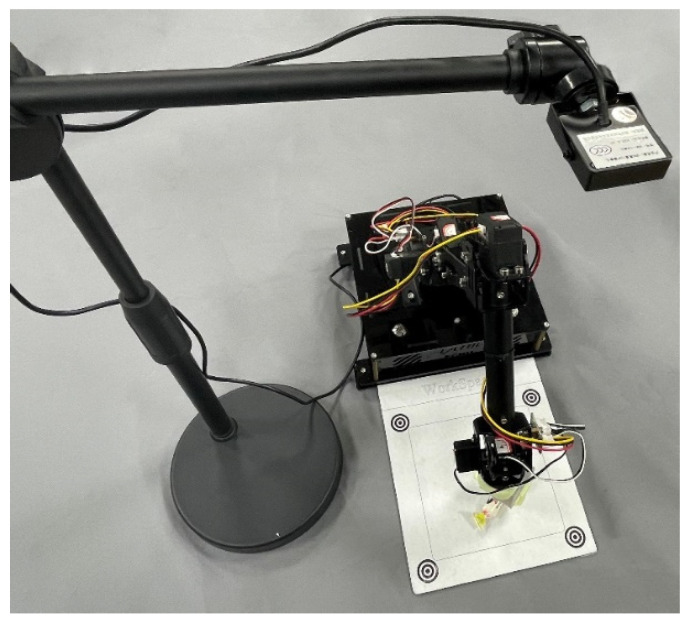
Vision pick of candy.

Vision pick refers to the action that iArm clamps the specified object in the workspace through the function of the vision module as shown in Figure 7. In this case, students are required to apply the vision module of iArm to clamp the specific candy in the workspace. 

Dimensions of Computational Thinking

Creative thinking: 

Students digitized the actual problem of a robotic arm clamping candy into a programmatic solution.

Problem-solving thinking: 

Students decompose the steps needed to solve the problem, which are mainly divided into two requirements of model building and model application and then decomposed into sub-requirements.

Algorithmic thinking: 

Students arrange the operations required for clamping to form an algorithm. In developing the model, they should photograph and label different types of candy, select the type of model, train the model, and assess it. In the model implementation, students should connect iArm, enable the visual function of iArm to detect and match the results. If the match is correct, iArm calculates the position and clips the target.

Critical thinking: 

When designing the algorithm and coding, students should consider how to optimize the model. For the accuracy of the vision pick, students should have the gripper clamp the middle of the candy to keep the clip stable. This requires students to make a comparison and judgment based on the performance of iArm.

Cooperative thinking: 

Considering the workload, this experiment is carried out through group cooperation to promote students’ cooperativity and communication skills.

#### 4.2.2. Experiment II: Multi-Arm Collaboration

Experiment Content

**Figure 8:**
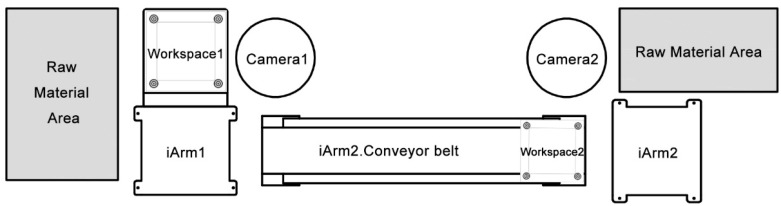
Placement of the equipment for multi-arm collaboration.

Multi-arm collaboration refers to the action that multiple robotic arms are driven by computer programs to collaboratively complete tasks. In this case, students are required to utilize two sets of iArm equipment, including two iArms, two workspaces, two cameras, and one conveyor belonging to iArm2, to move regular objects. Figure 8 shows the placement of two sets of iArm equipment in Experiment II. 

Dimensions of Computational Thinking

Creative thinking: 

Students complete multi-arm collaboration through programming and innovate the code according to their cognition, which promotes the improvement of their creative thinking.

Problem-solving thinking: 

Students summarize the subproblems in the task, that is, iArm1 puts the object on the conveyor belt, the conveyor belt transports the object to iArm2, and finally iArm2 clips the object to the raw material area.

**Figure 9:**
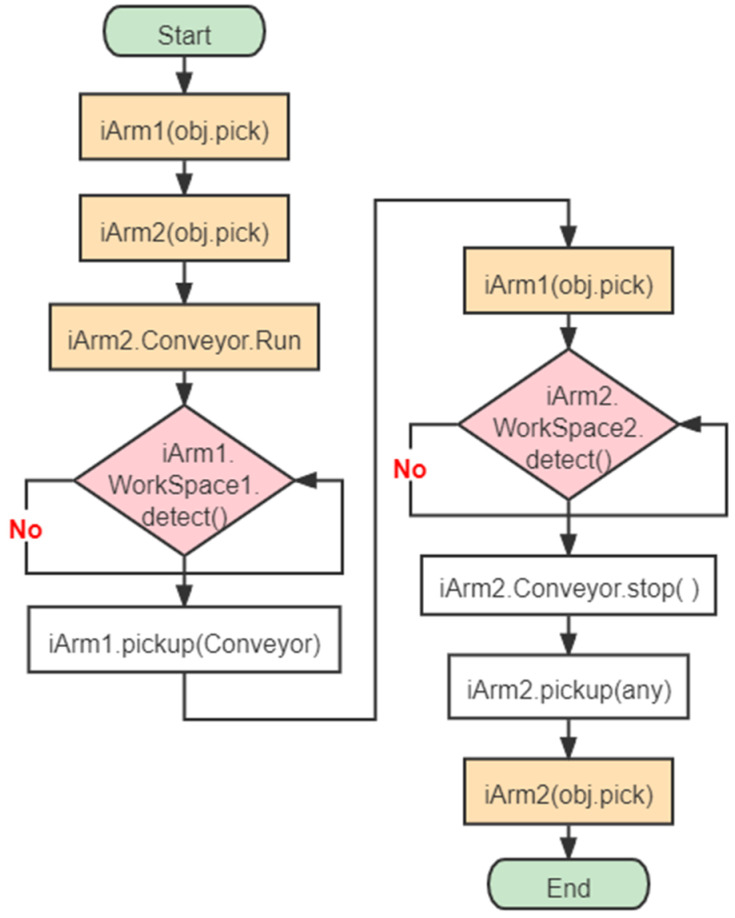
Key steps of the program in multi-arm collaboration.

Algorithmic thinking: 

Students refine the subproblems, design algorithms that fit the experiment, and implement the program. The major steps that students are expected to program are presented in Figure 9. iArm1 and iArm2 are pre-entered into the vision pick state and the conveyor belt is pre-operated. iArm1 picks the object to the conveyor belt if it detects the specified object. iArm2 detects the workspace2 and keeps the belt running until the object fits the pick requirement. Finally, iArm2 clips the object into the raw material area.

Critical thinking: 

Better ways should be considered to optimize the program, for example, markers attached to the conveyor belt are prone to incorrect recognition due to pattern noise. Students should consider which way can filter out errors and improve the accuracy of iArm2.

Cooperative thinking: 

Students are recommended to work in groups to discuss subproblems, including image recognition, device control, etc. In the collaboration, they will hone their communication skills and realize that harmonious teamwork leads to the high efficiency of task completion.

#### 4.2.3. Integrated Experiment: Industry 4.0 Simulation

Experiment Content

This experiment integrates Experiment I with Experiment II. AiTank, another robot for programming training developed by our team, is incorporated into the situation. A workspace board is also installed in front of the AiTank and grayscale sensors are respectively assembled on iArm2 and AiTank. It puts forward higher programming requirements to the users for not only applying the key steps of candy pick to multi-arm collaboration but also coordinating two iArms with the AiTank. 

**Figure 10:**
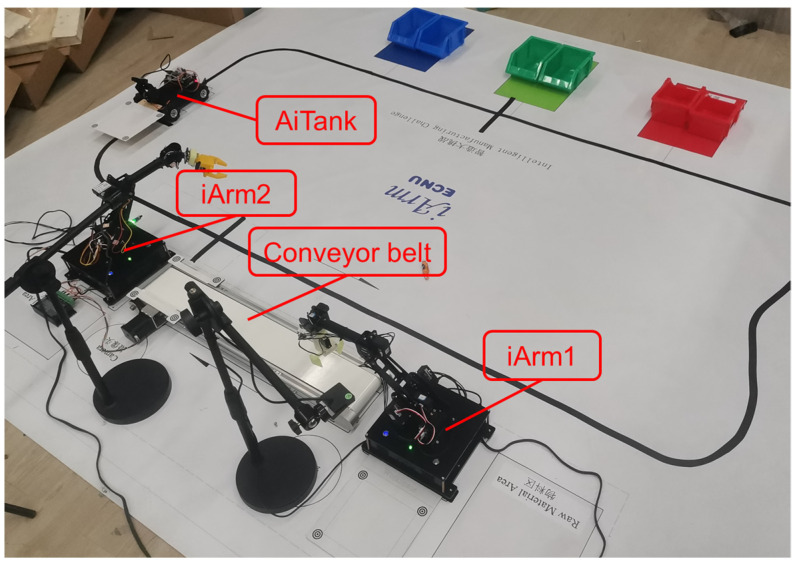
Placement of the equipment for Industry 4.0 simulation.

In Figure 10, the black line represents the trajectory that AiTank needs to patrol, and the black line vertical to the trajectory represents where AiTank needs to stop. Three color areas simulate the unloading area in industrial production. In this case, AiTank needs to transport the candies that iArm2 has placed on its workspace board to the unloading area and go to the next candy.

Dimensions of Computational Thinking

Creative thinking: 

The experiment requires students to simulate an Industry 4.0 scenario by the way of programming. They will need to incorporate what they have learned in previous experiments in this experiment. Due to the introduction of new tools, they should combine the AiTank into their programs creatively.

Problem-solving thinking: 

Students should extract the main parts of the experiment, including the vision pick in Experiment I, multi-arm collaboration in Experiment II, and AiTank’s patrol line control.

Algorithmic thinking: 

The experiment builds on the previous experiments and incorporates AiTank to train students in algorithmic thinking. AiTank has two states, among which State A is that the car keeps moving when nothing is detected on its workspace board, and it stops when the sensor detects the stop line; State B is that the car keeps moving when something is on its workspace and the car stops when the sensor detects the stop line. The states of iArm2 and AiTank interact with each other. iArm2 only calls the picking method when the grayscale sensor is high, that is, it detects the AiTank in front of it. 

Critical thinking: 

Students need to make appropriate adjustments to the position and power of the sensor on iArm2, as well as think about how to optimize the program to make it more efficient.

Cooperative thinking: 

The experiment is difficult for some high school students. Therefore, they will be aware of the importance of good cooperation when working in groups.

### 4.3. Assessment of iArm in Education

The curriculum has been implemented in a high school in Shanghai. 13 students participated in the curriculum, including freshmen and sophomores. They were divided into four groups. The first group was sophomores, and the second, third, and fourth groups were freshmen. Freshmen have no programming foundation and sophomores have a programming foundation. 

The study evaluates the application effect of iArm in education from the perspective of computational thinking. The evaluation is divided into the formative evaluation and the summative evaluation. According to the content of the course, we designed the evaluation table of students’ computational thinking ability as the standard of formative evaluation. In addition, the study issued a computational thinking questionnaire as the pre-test and post-test questionnaire to make a summative assessment.

#### 4.3.1. Assessment of Students’ Work

Students’ work was assessed from abstraction, algorithm, optimization, test, and generalization in computational thinking. Three learning projects, namely Linux Control, Robotic Arm Palletizing, and Color-based Pick, were selected from the preliminary, intermediate, and advanced stages for evaluation. The specific content of the evaluation standard is shown in Table 3.

**Figure 11:**
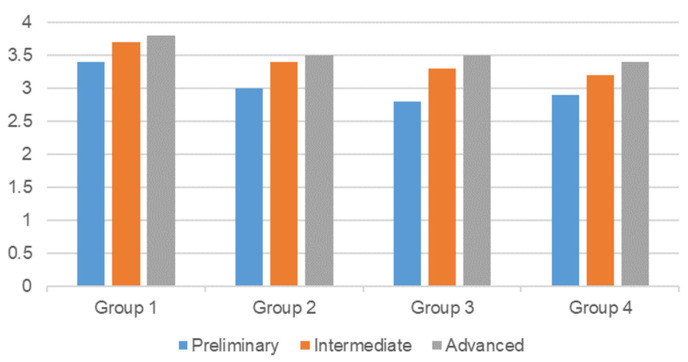
Average scores for each group at different stages of computational thinking.

Table 3 was adopted to evaluate the level of computational thinking of the work of each group of students. The results, as shown in Figure 11, indicated that all groups improved their computational thinking in the aspects of problem abstraction, algorithm design, iteration optimization, test and correction, and generalization during the course. It is worth noting that freshmen showed a greater improvement in computational thinking than sophomores. Freshmen went from 3, 2.8, and 2.9 to 3.5, 3.5, and 3.4. Sophomores went from 3.4 to 3.8.

#### 4.3.2. Assessment of Computational Thinking Questionnaire

**Figure 12:**
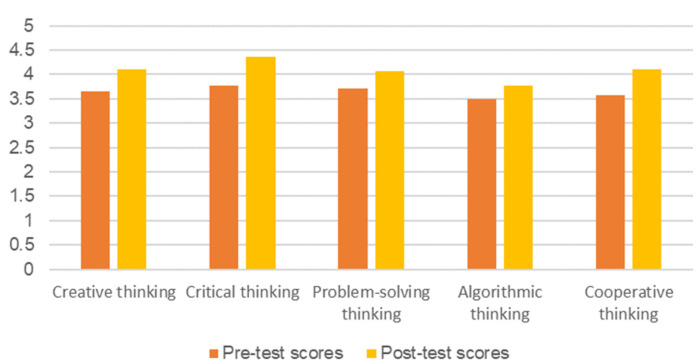
Pre-test and post-test average scores of students’ computational thinking ability.

A computational thinking questionnaire (Cronbach’s α = 0.895, N of Items = 23) was issued as a pre-test and post-test to analyze the changes in students’ computational thinking levels. The questionnaire is divided into five dimensions: creative thinking, critical thinking, problem-solving thinking, algorithmic thinking, and cooperative thinking. To explore the changes in computational thinking, we compare the average scores of the results shown in Figure 12. The results show that students’ computational thinking ability has improved in all five dimensions, especially their critical thinking (3.77 to 4.36) and cooperative thinking (3.58 to 4.10).

Over the course of one semester, students learned robotics and programming in educational practice containing various hands-on, technology-based activities which positively influenced the development of their creative skills [49]. The cultivation of algorithmic thinking resulted from building sequences of obtaining the intermediate results and the final goal, and planning the operation of actions based on their mathematical foundations [50]. Such a project-based learning curriculum using the educational robotic arm as the technology-enabled scaffolds can promote 21st-century skills that emphasize critical thinking and complex problem-solving thinking [51]. Students made reasonable inferences through the data presented in the entity tool, optimized their programs, and tested them. The improvement of all these five thinking literacies together fosters the progress of computational thinking.

## 5. Conclusions

This paper reviews existing educational robotic arms, the applications of arms, and computational thinking. The research has found that the development of educational robotic kits mainly focused on the robot itself and that the auxiliary devices should be refined. Commercial robotic arms are high-cost and closed-source, and thus are not suitable for wide application. Also, the educational application of robotic arms has not been concerned with the impact on computational thinking. These gave us the incentives to design an iArm kit for developing computational thinking ability. Furthermore, analysis was carried out to identify the effectiveness of the iArm kit in improving students’ computational thinking. Results show that after a semester of study, students have made progress in all aspects of computational thinking. The study fills in the gaps in the combination of the robotic arm and computational thinking. Due to the limited sample size, the generality of the results of this study remains to be verified. Also, the validity of the kit in other educational stages should be explored. Future work includes extending the availability of the iArm kit to other educational stages and exploring its effectiveness. Robotic arms can be a medium for students to learn and practice knowledge.

## Figures and Tables

**Table 1:** Standard D-H parameter.

Link	α_i_	a_i_	d_i_	θ_i_	Offset
L1	pi/2	0.0452	0.082	θ_1_	0
L2	0	0.1165	0	θ_2_	pi/2
L3	pi/2	0.01825	0	θ_4_	0
L4	pi/2	0	0.165	θ_4_	0
L5	−pi/2	0	0	θ_5_	0
L6	0	0	0.044	θ_6_	0

**Table 2:** Five-dimensional computational thinking development objectives.

Development Dimensions	Objectives Content
Problem Abstraction	Be able to specify the objectives and conditions of a problem for a given task; be able to abstract the problem, decompose it into some executable operational steps, and give concrete processes and methods for solving the problem.
Algorithm Design	Analyze and extract data, design an algorithm based on needs, describe the algorithm using a flowchart, and program with the appropriate algorithm.
Iteration Optimization	Use iterative thinking to analyze solutions to problems with some degree of optimization and be able to evaluate its rationality and completeness, and analyze the possibilities for optimization or improvement of the solution.
Test and Correction	Find bugs in the process of trying, verifying, and modifying, and then fix them by debugging the code.
Generalization and Application	Generate solutions to problems and apply them to other relevant problems in real life.

**Table 3:** The scale of computational thinking ability.

Stage	Aspect	Details
Preliminary stage (25 marks)	Abstraction (5 marks)	Control variables to realize the basic motion of the robotic arm (linear/ oblique motion).
	Algorithm (5 marks)	Algorithm implementation: Graphical programming Python programming Algorithm design: Obtain joint coordinates in learning mode Control variables in an endpoint coordinate system Shift the axis coordinates
	Optimization (5 marks)	Loop structure “repeat … times, do …”
	Test (5 marks)	Run the program, observe the movement of iArm, and modify the code according to the actual error.
	Generalization (5 marks)	Summarize three algorithms and two programming methods for controlling manipulator motion.
Intermediate stage (25 marks)	Abstraction (5 marks)	Plan the path to realize simple applications of robotic arms (palletizing, writing)
	Algorithm (5 marks)	Algorithm implementation: Graphical programming Python programming Algorithm design: “point-to-point” path planning Single path planning (palletizing for once, single-stroke painting) Multiple path planning (palletizing for multiple times, multiple strokes painting)
	Optimization (5 marks)	Create variables and assign values; loop structure (while)
	Test (5 marks)	Run the program, observe the painting and palletizing, and modify the code according to the actual error.
	Generalization (5 marks)	Summarize methods of multiple path planning and be able to plan different movements according to the task requirements.
Advanced stage (25 marks)	Abstraction (5 marks)	Introduce a vision module to realize the artificial intelligence application of robotic arms (Color-based pick)
	Algorithm (5 marks)	Algorithm implementation: Graphical programming Python programming Algorithm design: Create workspace by visual calibration. Set parameters to call the visual_pick function. Plan the path, pick up the specific color block and place it in the corresponding area
	Optimization (5 marks)	Selection structure “if …”
	Test (5 marks)	Run the program, observe the effects of vision pick, and modify the code according to the actual error.
	Generalization (5 marks)	Summarize the method of picking different color blocks and be able to plan different paths according to task requirements.

## Data Availability

The data presented in this study are available from the corresponding author upon reasonable request.
